# Feasibility analysis of arterial CT radiomics model to predict the risk of local and metastatic recurrence after radical cystectomy for bladder cancer

**DOI:** 10.1007/s12672-024-00880-x

**Published:** 2024-02-19

**Authors:** Huawang Lv, Xiaozhou Zhou, Yuan Liu, Yuting Liu, Zhiwen Chen

**Affiliations:** Department of Urology, Southwest Hospital, Army Medical University (Third Military Medical University), Chongqing, 400038 China

**Keywords:** Bladder cancer1, Radical cystectomy2, Local or metastatic recurrence3, Arterial CT imaging4, Radiomics5, Retrospective studies6

## Abstract

**Purpose:**

To construct a radiomics-clinical nomogram model for predicting the risk of local and metastatic recurrence within 3 years after radical cystectomy (RC) of bladder cancer (BCa) based on the radiomics features and important clinical risk factors for arterial computed tomography (CT) images and to evaluate its efficacy.

**Methods:**

Preoperative CT datasets of 134 BCa patients (24 recurrent) who underwent RC were collected and divided into training (n = 93) and validation sets (n = 41). Radiomics features were extracted from a 1.5 mm CT layer thickness image in the arterial phase. A radiomics score (Rad-Score) model was constructed using the feature dimension reduction method and a logistic regression model. Combined with important clinical factors, including gender, age, tumor size, tumor number and grade, pathologic T stage, lymph node stage and histology type of the archived lesion, and CT image signs, a radiomics-clinical nomogram was developed, and its performance was evaluated in the training and validation sets. Decision curve analyses (DCA) the potential clinical usefulness.

**Results:**

The radiomics model is finally linear combined by 8 features screened by LASSO regression, and after coefficient weighting, achieved good predictive results. The radiomics nomogram developed by combining two independent predictors, Rad-Score and pathologic T stage, was developed in the training set [AUC, 0.840; 95% confidence interval (CI) 0.743–0.937] and validation set (AUC, 0.883; 95% CI 0.777–0.989). The calibration curve showed good agreement between the predicted probability of the radiomics-clinical model and the actual recurrence rate within 3 years after RC for BCa. DCA show the clinical application value of the radiomics-clinical model.

**Conclusion:**

The radiomics-clinical nomogram model constructed based on the radiomics features of arterial CT images and important clinical risk factors is potentially feasible for predicting the risk of recurrence within 3 years after RC for BCa.

## Introduction

Bladder cancer (BCa) is one of the most common malignant tumors in humans and the most common among all urological tumors [1]. Based on whether it invades the bladder muscle layer, BCa can be divided into muscle invasive bladder cancer (MIBC) and non-muscle invasive bladder cancer (NMIBC) [2, 3]. Radical cystectomy (RC) is one of the main treatments for nonmetastatic muscle invasive bladder cancer (NMMIBC) or high risk non-muscle invasive bladder cancer (HRNMIBC) [4]. Generally, the prognosis of patients with MIBC after RC is generally worse than that of patients with NMIBC. Moreover, there are significant individual differences in the prognosis of RC for BCa of the same tumor stage determined by tumor heterogeneity [5]. Studies have shown that, despite RC treatment, local or metastatic recurrence rates of patients with BCa are 22–47% [6]. In these patients, the prognosis is often very unsatisfactory, and early and accurate prediction of recurrence risk and individualized treatment and follow-up management should be improved [7].

Computed tomography (CT) is the most commonly used preoperative evaluation in patients with BCa to localize the tumor and determine its number, size, degree of invasion of surrounding tissue, and presence of lymph node metastases or distant metastases. However, tumor heterogeneity cannot be reliably assessed visually. Radiomics refers to the extraction of many medical image texture features from raw image data and converting them into quantitative, high dimensional data [8]. Recent literature has shown that the radiomics features of CT or magnetic resonance imaging (MRI) have a significant impact on predicting the pathologic grade, lymph node metastasis, and overall survival of BCa [9–11]. At present, scholars have begun to explore the prediction of recurrence of BCa after surgery but have not yet elaborated on the prognosis of patients after RC for BCa [12, 13]. The purpose of this paper was to establish a nomogram related to radiomics to further explore the prediction of the risk of local and metastatic recurrence after RC for BCa.

Based on these findings, we hypothesized the following: (1) the nomogram can extract high throughput radiomics features that are highly correlated with BCa recurrence from raw CT images; (2) the nomogram model established by radiomics characteristics combined with important clinical factors can accurately and efficiently predict the recurrence of BCa.

## Materials and methods

### Patients

The retrospective study was approved by an institutional ethics review board and exempted patients from the need for informed consent, the approval numbers and dates of the Institutional Review Borad are (B) KY2022238 and December 30, 2022, respectively. In total, 134 patients who underwent RC at the Southwest Hospital of Army Medical University from June 2014 to June 2019 and were diagnosed with postoperative pathological confirmation of BCa were retrospectively enrolled. The inclusion criteria were as follows: (1) underwent RC and was pathologically confirmed as BCa; (2) CT scan was performed 1 month before RC, and the images were clear; and (3) complete clinicopathological data were available. The exclusion criteria were as follows: (1) CT scan of poor quality, mainly manifested as unclear display of bladder tumors caused by artifacts or other factors; (2) other treatment, including chemotherapy or immunotherapy, prior to CT scan; and (3) incomplete clinicopathological data or loss to follow-up.

### Research method

#### Clinicopathological data and follow-up contents

Information of patients in the First Affiliated Hospital of Army Medical University from June 2014 to June 2019 was retrospectively collected, and the clinical data mainly included gender, age, tumor number, tumor size and grade, pathologic T stage, lymph node stage and histology type. All patients were followed every 3 months for the first year postoperatively and then every 6 months in years 2 and 3. The most important postoperative follow-up test to determine if BCa has returned is a CT scan. If the examination results show that the bladder tumor reappears in the urethra and pelvic cavity after RC; Or distant sites, such as the lung, bone, brain or liver, are considered for local or metastatic recurrence of BCa. Relapse-free survival (RFS) was recorded. After 3 years of follow-up, 24 cases of BCa recurrence were confirmed, which were defined as the recurrence group, and the rest were classified as the no recurrence group.

#### CT image acquisition

Prior to RC, all patients had a CT examination (Siemens Healthineers Somatom Sensation 64). The CT scan parameters were as follows: tube voltage, 120 kV; automatic tube current; detector collimation, 64 × 0.6 mm; matrix, 512 × 512; and rack rotation time, 500 ms. The intravenous contrast agent iohexol (350 mg I/ml) was scanned by intravenous injection of the elbow at an injection rate of 3–3.5 ml/s, followed by a flushing tube with 20 ml of saline. After collecting the localization images, arterial and venous images were obtained at 25 s and 60 s, respectively, and arterial phase images with a thickness of 1.5 mm were selected for feature extraction.

#### Region of interest (ROI) delineation and radiomics feature extraction

The first senior reader in genitouriturological imaging (patients whose clinicopathological data and follow-up results were unknown) reviewed all CT scans and manually cut and mapped the ROI of the entire tumor layer by layer using the medical imaging software 3D Slicer (version 4.13.0 http://www.slicer.org). Another reader with extensive experience in urogenital imaging (who was also unaware of the clinicopathological data and follow-up results of the patients) reviewed all the ROIs assigned by the first reader and randomly selected 45 lesions for resegmentation. Python (version 3.7.1) and Jupyter Notebook (version 5.7.4) were combined to perform feature extraction on ROIs that had already been cut. The main steps were as follows:

- 1. Image preprocessing: The plug in Pyradiomics (version 3.0.1) was used to resample all images, the parameters binWidth were set to 25, the parameters resampledPixelSpacing were set to 1.0 mm, Then, the image nonlinear intensity transformation and wavelet transform are performed on the original CT image to reduce computational interference and improve feature recognition ability.
- 2. Feature extraction and calculation: Based on the original CT image and processing, 1316 radiomics features were extracted from each ROI: firstorder features that describe single pixels of the image; shape features that describe the geometric characteristics of the ROI; and texture features that reflect the homogeneous phenomenon of vision in the image, including 252 firstorder features, 14 shape features, 336 gray-level cooccurrence matrix (GLCM) features, 224 gray-level run length matrix (GLRLM) features, 224 gray-level size zone matrix (GLSZM) features, 70 neighborhood gray tone difference matrix (NGTDM) features, and 196 gray-level dependence matrix (GLDM) features.

#### Local and metastatic recurrence-related feature selection and radiomics scoring model establishment

**Fig. 1:**
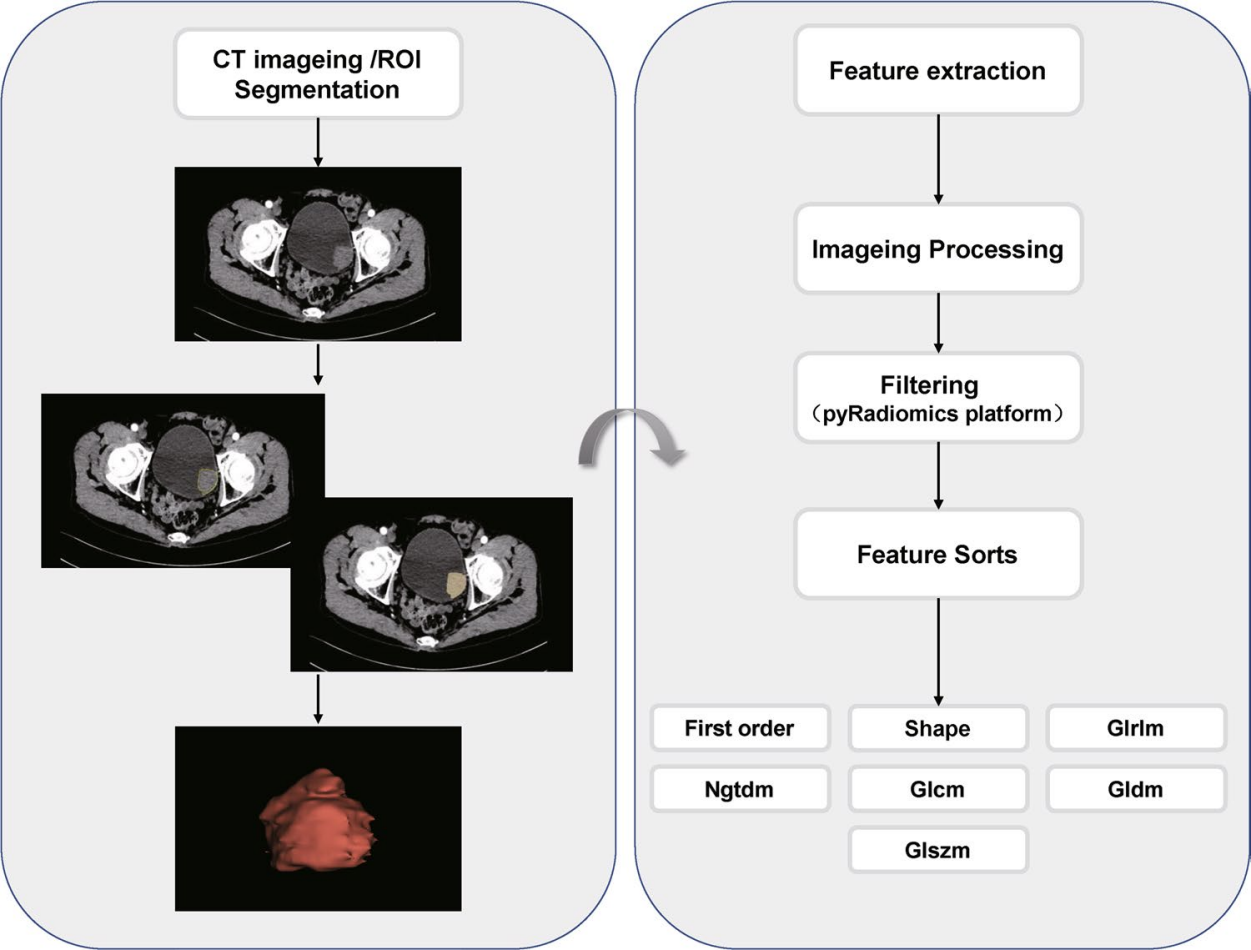
Workflow for extraction and screening of radiomic features

- 1. Feature selection: First, to evaluate the repeatability of radiomics feature extraction, we calculated the interclass correlation coefficient (ICC) of the feature on the basis of the previous two experts' work and selected features with ICC>0.75. Subsequently, in training set, to maintain the comparability of different features and reduce the imbalance in the importance of each feature caused by the differences in the mean and variance of each feature, we normalized the features of the Z score: z= xi-μ/δ. Finally, to reduce the difficulty of model learning and data noise in the later stage, the coefficient of association and analysis of variance were used for feature selection. After removing other redundant and useless features, the features of p<0.05 were retained as an effective factor for the next feature selection.
- 2. Radiomics Scoring Model Establishment: To solve the overfitting problem of high dimensional data, improve the performance of the model, and select the most effective prognostic features, the LASSO regression algorithm was used to select the deviation of the measurement index, and the optimal feature was selected after 10-fold cross verification. In the training group, a logistic regression model with radiomics scoring was constructed based on the selected radiomics features to obtain the regression coefficients. Then, a linear combination of these selected features and their corresponding regression coefficients was used to calculate the Rad-Score for each patient. Then, validation was performed in the validation group. The score value was used for subsequent analysis. The study design for radiomics is shown in Figure 1.

#### Establishment of radiomics-clinical model and efficacy assessment

First, univariate analysis was carried out, and the statistically significant variable P<0.05 was retained as a clinical risk factor, combined with Rad-Score and clinical variables. The multivariate logistic regression model was used for analysis, and P <0.05 was further determined to be an independent risk factor. Then, the radiomics clinical nomogram predictive type related to BCa recurrence was established. The training set and validation set calibration curves and receiver operating characteristic curves (ROC) were used to evaluate the performance. Finally, according to the median recurrence risk score of the line chart model training dataset, Decision curve analyses (DCA) was established to further evaluate the clinical benefit of the radiomics-clinical model compared with the radiomics model and the clinical model alone.

### Statistical analysis

SPSS 26.0 software (IBM) was used for univariate and multivariate analyses to study the clinical risk factors and independent risk factors among the cohort. A t test was used for measurement data, a chi-square test was used for counting data, and single factor analysis of variance was used for preliminary feature screening. In this study, R statistical software version 4.2.2 (https://www.r-project.org/) was used for ICC calculation, model building and performance evaluation, and survival curve drawing. The R packages used included “glmnet,” “rms,” “pROC,” “rmda,” and “survival.” P<0.05 indicates statistical significance.

## Results

### Patient characteristics

Overall, 134 patients with BCa were enrolled in this study. All patients underwent RC and pelvic lymph node dissection followed by urinary diversion (Briker or orthotropic ileal bladder surgery). Among them, 119 were males, and 15 were females, with a mean age of 64.4 ± 9.7 years. During the 3-year follow-up, patients were divided into a recurrence group (n=24) and a no recurrence group (n=110) according to whether they relapsed. The median RFS of patients with relapse was 12 months. Comparisons of gender, age, tumor number, tumor size, grade, pathologic T stage, lymph node stage and histology type between the two groups are shown in Table 1.

**Table 1:** Demographics and Clinical Characteristics of Patients in the training set and validation set

Factors	Overall (N = 134)	Training set, n (%)			Validation set, n (%)		
		No recurrence (N = 74)	Recurrence (N = 19)	P-value	No recurrence (N = 36)	Recurrence (N = 5)	P-value
Gender, n (%)				0.310			0.501
Female	15 (11.2%)	4 (5.4%)	3 (15.8%)		8 (22.2%)	0 (0%)	
Male	119 (88.8%)	70 (94.6%)	16 (84.2%)		28 (77.8%)	5 (100%)	
Age, n (%)				0.917			0.958
< 60	37 (27.6%)	19 (25.7%)	4 (21.1%)		12 (33.3%)	2 (40.0%)	
≥ 60	97 (72.4%)	55 (74.3%)	15 (78.9%)		24 (66.7%)	3 (60.0%)	
Number of tumors, n (%)				0.990			0.029
Single	89 (66.4%)	48 (64.9%)	12 (63.2%)		28 (77.8%)	1 (20.0%)	
Multiple	45 (33.6%)	26 (35.1%)	7 (36.8%)		8 (22.2%)	4 (80.0%)	
Size of tumors, n (%)				0.045			0.776
< 3	52 (38.8%)	35 (47.3%)	3 (15.8%)		13 (36.1%)	1 (20.0%)	
≥ 3	82 (61.2%)	39 (52.7%)	16 (84.2%)		23 (63.9%)	4 (80.0%)	
Grade, n (%)				0.070			0.308
High	93.0 (69.4%)	57 (77.0%)	19 (100%)		24 (66.7%)	5 (100%)	
Low	41.0 (30.6%)	17 (23.0%)	0 (0%)		12 (33.3%)	0 (0%)	
pT stage, n (%)				< 0.001			0.037
≥ 2	62 (46.3%)	27 (36.5%)	16 (84.2%)		14 (38.9%)	5 (100%)	
< 2	72 (53.7%)	47 (63.5%)	3 (15.8%)		22 (61.1%)	0 (0%)	
pN stage, n (%)				0.015			0.509
N0	125 (93.3%)	72 (97.3%)	15 (78.9%)		34 (94.4%)	4(80.0%)	
N1/N2	9 (6.7%)	2 (2.7%)	4 (21.1%)		2 (5.6%)	1 (20.0%)	
Histology type, n (%)				0.992			0.038
Urothelial carcinoma	129 (96.3%)	73 (98.6%)	19 (100%)		34 (94.4%)	3 (60%)	
Non-urothelial carcinoma	5 (3.7%)	1 (1.4%)	0 (0%)		2 (5.6%)	2 (40%)	
RFS (months)				< 0.001			< 0.001
Median [Min, Max]	60 [3, 96]	51 [36, 95]	13 [3, 35]		60 [36, 96]	13 [6, 29]	

### Selection of radiomics features and establishment of radiomic model

**Fig. 2:**
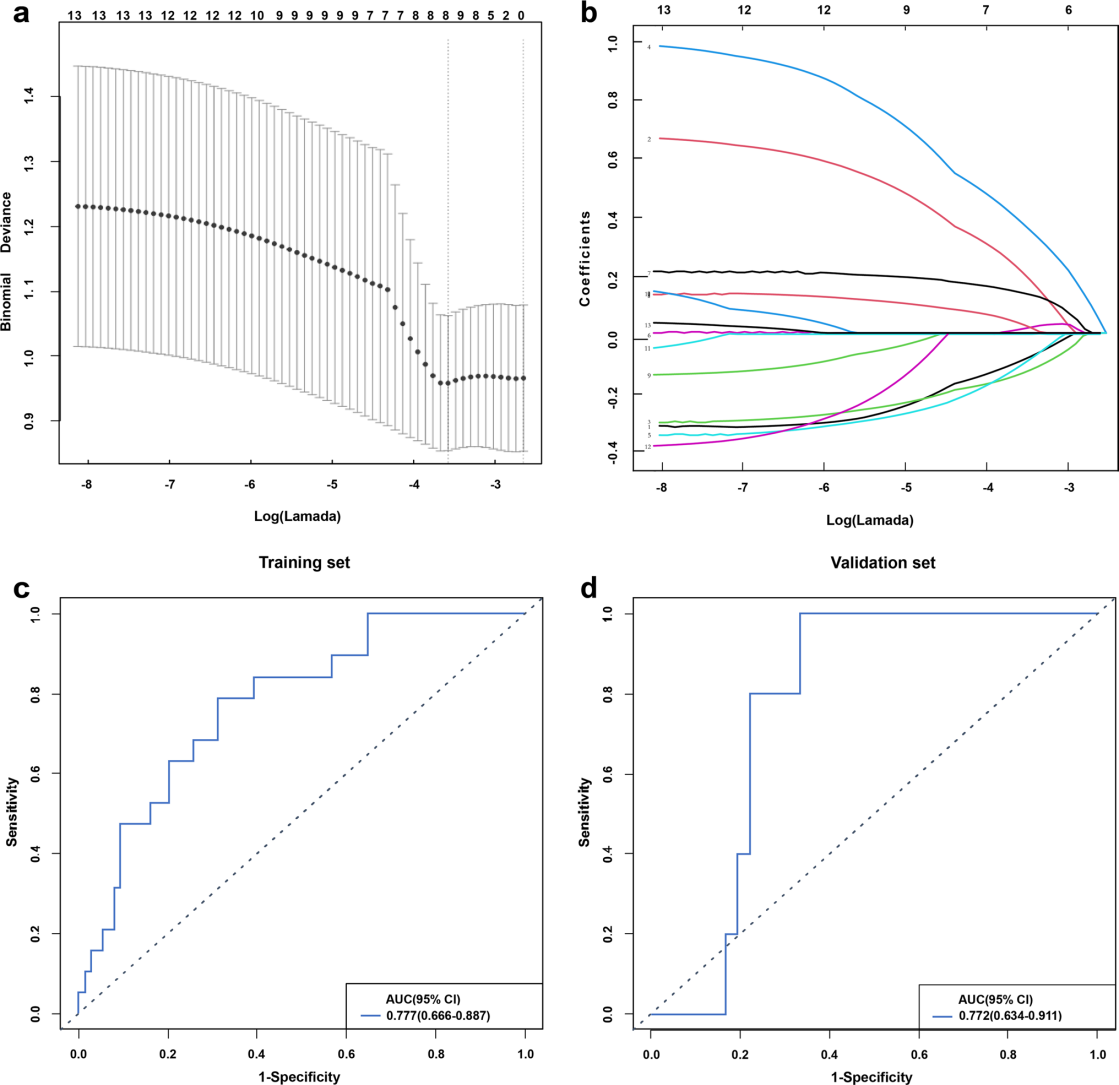
**a** The LASSO model is selected by tenfold cross verification based on minimum criteria; **b** LASSO coefficient distribution map for 8 texture features; **c** and **d** are ROC curves of the training set and validation set radiomic features

In this study, 1316 radiomics features were extracted, among which the average features of ICC>0.75 showed good agreement between the two groups of observers, and 1076 features of ICC>0.75 prepared the following feature selection. The correlation threshold of 0.9 was selected for correlation coefficient analysis, and the 13 best features with a P value of <0.05 were then screened out by analysis of variance. Each feature data point was standardized, and 8 features were screened out after 10-fold cross verification (Fig. 2a, b) using LASSO regression algorithm. Finally, they were established as logistic regression models for radiomics. The model showed an AUC of 0.777 (95% CI 0.666–0.887) for the training set and 0.772 (95% CI 0.634–0.911) for the validation set (Fig. 2c, d). Based on the independent variable coefficients and cutoff values of the radiomics model, the regression coefficients were obtained. In the training set, the 8 retained features were linearly combined by the corresponding coefficient weighting to obtain the calculation formula of Rad-Score, which could quantify the radiomic features and predict the recurrence risk.

### Establishment of a radiomic-clinical model and its efficacy assessment

**Fig. 3:**
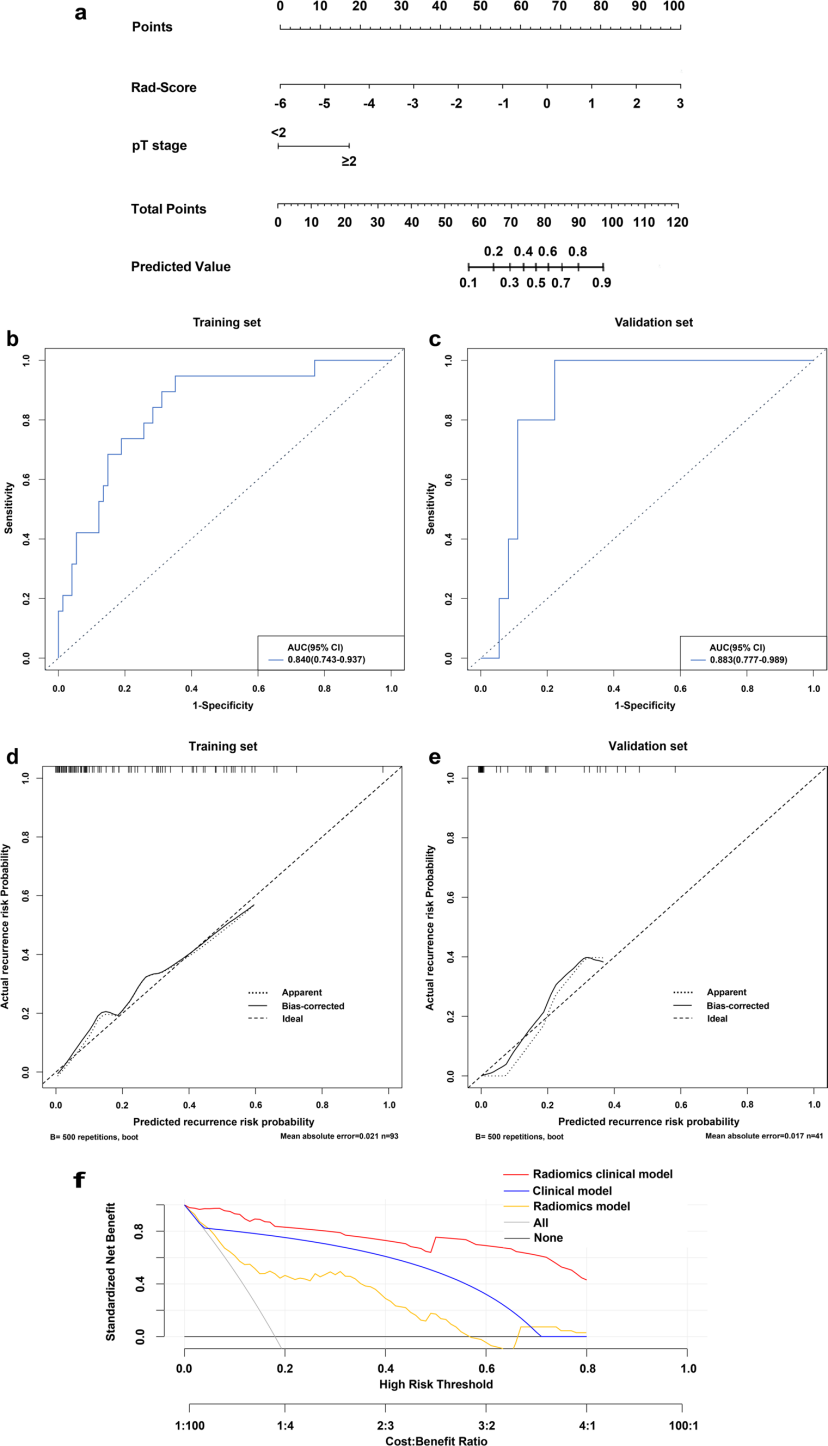
**a** Nomogram constructed based on pathologic T stage and Rad-Score; ROC curves and calibration curves of the radiomics-clinical model in training **b**, **d** and validation **c**, **e** sets; **f** DCA for the radiomics-clinical model nomogram

**Fig. 4:**
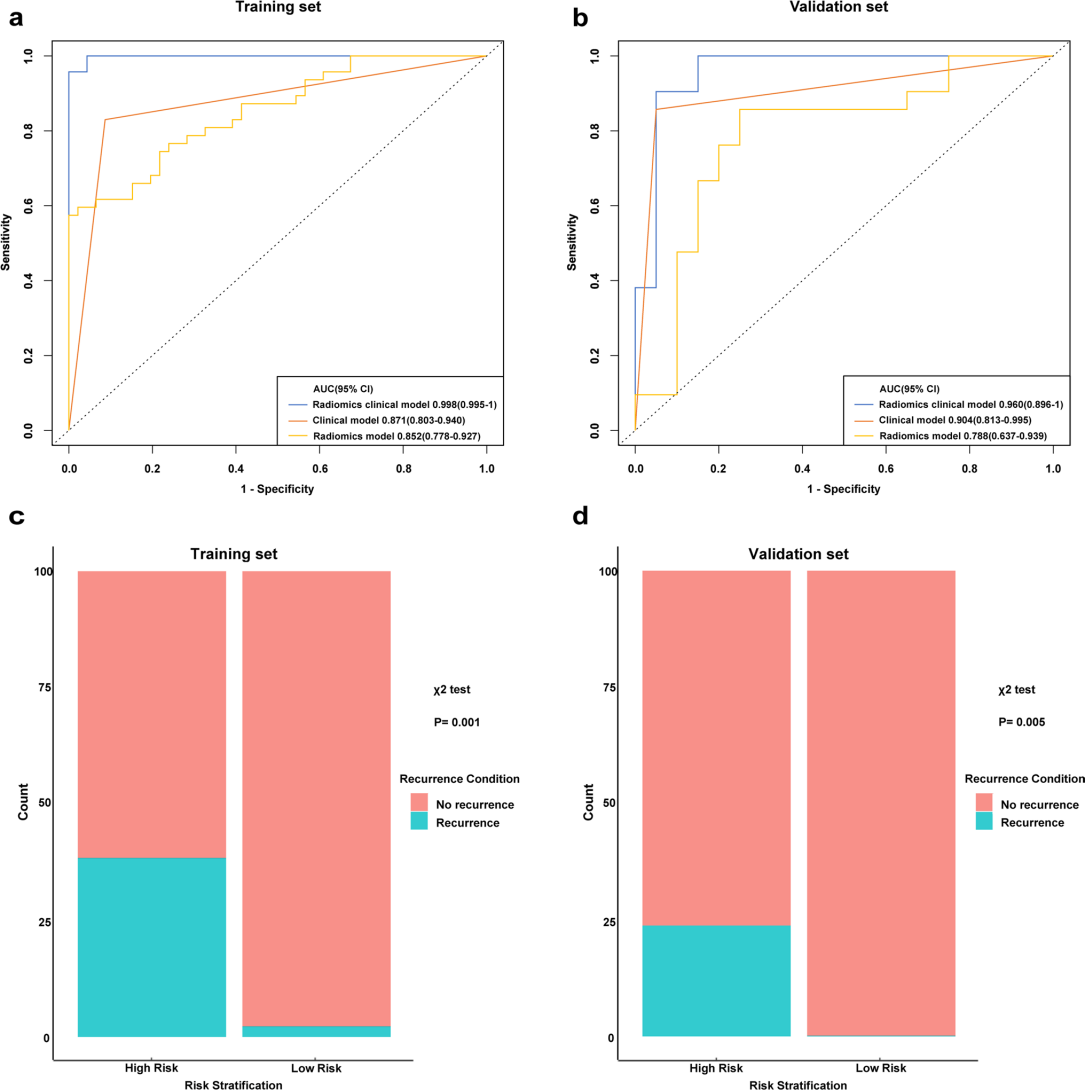
ROC curves and chi-square plots of different risks and models in training **a**, **c** and validation **b**, **d** sets

**Fig. 5:**
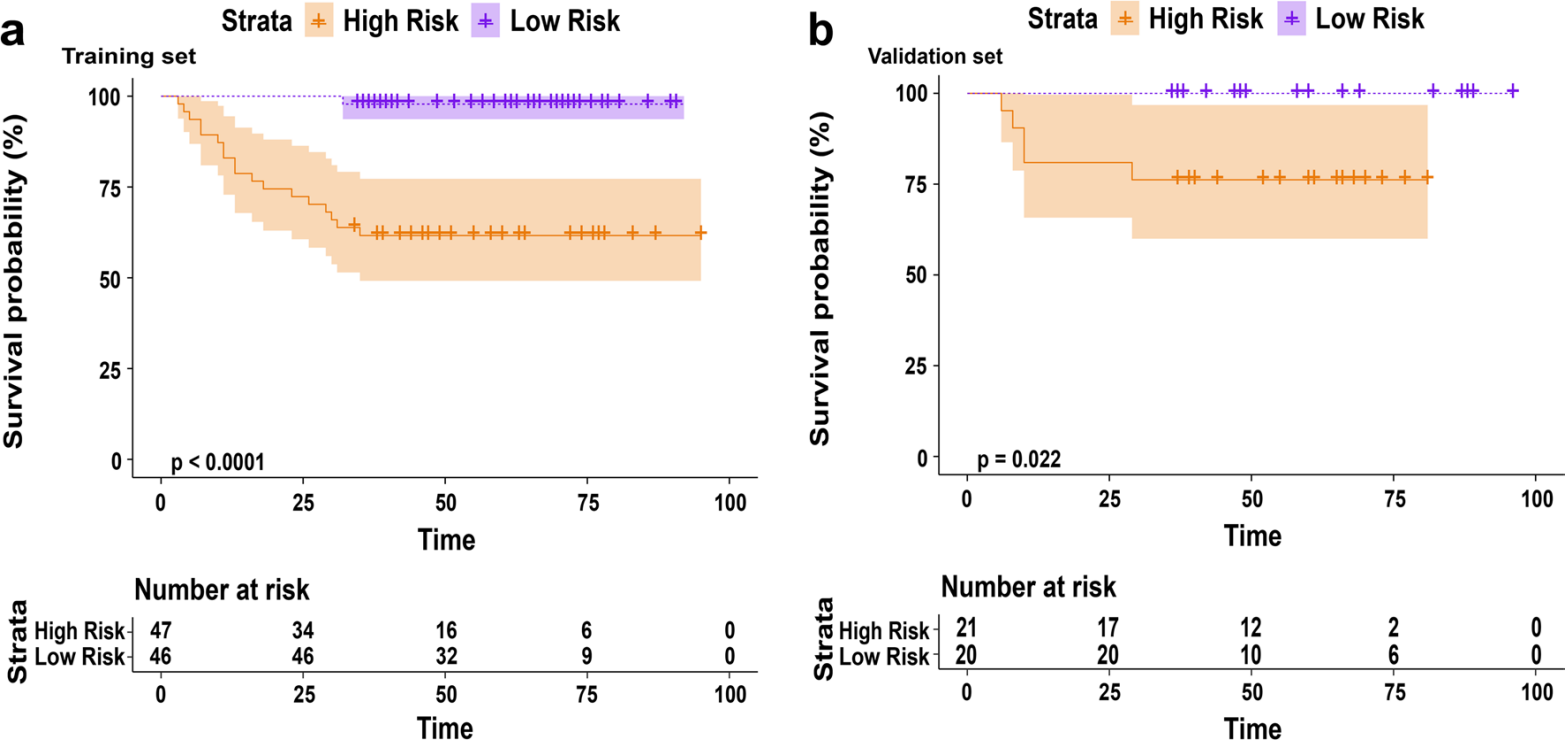
Kaplan–Meier plots of the RFS for training set (**a**) and validation set (**b**)

Univariate and multivariate analyses were performed based on the Rad-Score and important clinical factors of the patients in the training set, in which tumor size, pathologic T stage, lymph node stage and Rad-Score were identified as clinical risk factors (P <0.05). Meanwhile, in the multivariate analysis based on clinical risk factors, pathologic T stage (OR 0.176, 95% CI 0.036–0.653 P=0.015) and Rad-score (OR 2.933, 95% CI 1.336–7.951 P=0.018) were still identified as independent prognostic factors, as shown in Table 2. Combined with the Rad-score and pathologic T stage, a radiomics-clinical nomogram model for predicting local or metastatic recurrence after RC in BCa patients was established (Fig. 3a). The ROC curves of the model showed that the AUCs of the training set and the validation set were 0.840 (95% CI 0.743-0.937) and 0.883 (95% CI, 0.777-0.989), respectively, the accuracies were 0.817 and 0.878 (Fig. 3b, c), respectively, and the calibration curves of the training set and the validation set performed well, showing that the model can predict the recurrence rate of BCa within 3 years after RC in good agreement with the actual situation (Fig. 3d, e). According to the nomogram, if the patient is pathologic T stage≥2 and the Rad-Score is -2, the scores will be close to 17.5 and 45, respectively, and the “total score” is 62.5 points, which corresponds to a “recurrence risk” of approximately 0.18 points. The patients were divided into a low risk recurrence group and a high risk recurrence group according to the median recurrence risk in the training set (0.12). The decision curves of different models in the cohort are shown in Fig. 3f, DCA shows that the clinical utility performance of the radiomics-clinical model is higher than that of the clinical model and the radiomics model. As shown in Fig. 4, the performance of recurrence risk stratification was quantitatively evaluated, and the comprehensive prediction performance of the radiomics-clinical model was significantly better than that of the clinical model and the radiomics model, as shown in Table 3. Based on nomogram recurrence risk stratification, Kaplan-Meier (KM) plots of RFS in the first 3 years after RC in the low risk recurrence group and high risk recurrence group were plotted, as shown in Fig. 5.

**Table 2:** Univariate and multivariate regression analyses of the indicators for recurrence prediction in the training set

	Univariate		Multivariate	
Factors	OR (95% CI)	P	OR (95% Cl)	P
Gender	0.305 (0.061–1.671)	0.144		
Age	1.295 (0.409–4.971)	0.678		
Number of tumors	1.076 (0.362–3.021)	0.890		
Size of tumors	4.786 (1.444–21.829)	0.020	1.774 (0.415–9.294)	0.456
Grade	2.538 (0.637–16.949)	0.243		
pT stage	9.259 (2.786–43.478)	0.001	5.682 (1.531–27.778)	0.015
pN stage	9.600 (1.716–73.979)	0.013	3.924 (0.515–39.299)	0.201
Histology type	4.056 (0.155–105.917)	0.330		
Rad-Score	3.875 (1.852–9.935)	0.002	2.933 (1.336–7.951)	0.018

**Table 3:** Different models evaluated the diagnostic performance of relapse risk stratification

	Training set				Validation set			
Model types	AUC	95% CI	Harrell’s C-index	p-value	AUC	95% CI	Harrell’s C-index	p-value
Radiomics clinical model	0.998	0.995–1	0.957	< 0.05	0.960	0.896–1	0.855	< 0.05
Clinical model	0.871	0.803–0.940	0.743	< 0.05	0.904	0.813–0.995	0.807	< 0.05
Radiomics model	0.852	0.778–0.927	0.574	< 0.05	0.788	0.637–0.939	0.607	< 0.05

## Discussion

Recurrence after RC is one of the main factors affecting the prognosis of BCa [14]. At present, the most important method for diagnosing BCa is cystoscopy. Imaging plays an irreplaceable role in the early diagnosis and evaluation of treatment effects in patients and has obvious noninvasive advantages over endoscopy [15–18]. Pathological grade is still the most critical factor affecting the treatment and prognosis of BCa, but with the rise of radiomics applications in oncology [19], extracting high throughput information based on raw images may be able to quantify tumor heterogeneity earlier [20, 21]. Therefore, noninvasive tools for predicting recurrence after RC for BCa appear to be valuable in clinical decision-making.

To date, several relevant studies have reported the application value of CT radiomics in predicting the prognosis after BCa surgery, among which Piotr Woznicki et al. found that radiomics features based on preoperative CT scans had prognostic value in predicting overall survival before RC in a study of 301 BCa patients who underwent RC and pelvic lymphadenectomy. Among them, the AUC of the clinical model was 0.761, and the AUC of the radiomics model was 0.771, which suggests that the predictive performance of the radiomics model is comparable to that of the verified clinical parameters [11]. Qian et al. reported that radiomics features extracted from multistage CT images combined with important clinicopathological risk factors can predict the recurrence of BCa 2 years after surgery, but the recurrence of BCa after RC was not included separately in the systematic study [12, 13]. Therefore, whether CT radiomics combined with clinical factors can predict local recurrence or metastatic recurrence after RC for BCa needs to be further verified.

In this study, a radiomics-clinical nomogram model based on preoperative CT extraction of high throughput radiomics features and important clinical risk factors was initially explored for individualized BCa recurrence risk stratification after RC.

Among the important clinical factors selected, our univariate analysis showed that tumor size, pathologic T stage, and lymph node stage were strongly associated with tumor recurrence after RC in patients with BCa and that these factors influenced the prognosis of patients, consistent with previous studies [22, 23]. However, tumors are heterogeneous in individual patients, and the TNM staging system used to predict long term survival is not entirely accurate [24–26]. Interestingly, based on high throughput data normalization and the lasso regression algorithm, eight radiomics texture features with the highest correlation with tumor recurrence after RC in patients with BCa were finally included, including one firstorder feature, four GLSZM features, one GLCM feature, one GLDM feature and one GLRLM feature. In recent years, they have been extensively studied [27, 28]. Preliminary evidence suggests that these features may be a good characterization of histoheterogeneity and histopathological differences among patients with BCa [29]. Multivariate analysis combining tumor size, pathologic T stage, lymph node stage and Rad-Score showed that pathological T stage and Rad-Score were independent risk factors for tumor recurrence after RC. Based on both, we developed a radiomics-clinical nomogram model for recurrence risk stratification. The results showed that the AUC (95% CI) of the training and validation sets of the nomogram model was 0.840 (0.743–0.937) and 0.883 (0.777–0.989), respectively, which was significantly higher than that of the radiomics model, indicating that the radiomics- clinical model was superior to the radiomics model in predicting recurrence after RC in patients with BCa. The calibration plot confirmed that the predicted performance is reliable and valid. DCA also clearly showed that, when the risk threshold is greater than 0.1, the composite model has more net benefits than the clinical model alone or the radiomics model.

According to the nomogram, when pathologic T stage was less than 2 and Rad-Score was -0.5717195401, the minimum value reaching the predicted value was less than 0.1. When pathologic T stage was more than 2, the Rad-Score was 2.336809971, the maximum predicted value is greater than 0.9. The recurrence risk group was divided according to the median predicted value of the training set's nomogram model, that is, the median recurrence risk score. the comprehensive comparison of various factors showed that the radiomics-clinical model is still better than the other two models. Chi-square test results showed a close correlation between recurrence risk stratification and true recurrence.

KM map analysis showed that RFS of patients with different recurrence risk groups were also different. The performance of radiomics-clinical nomogram is valuable for the estimation of RFS. In summary, the nomogram scoring system of this study may serve as a more effective tool to accurately and rapidly assess the risk of recurrence after RC through simple computational methods, enabling early individualized treatment of BCa patients.

Our current research still has some unanswered questions. First, this was a single center study with a limited sample size, which requires additional multicenter data samples and external validation. Second, due to the incompleteness of the data in the single institutional database, possible predictors, such as tumor location and radical urinary diversion, were not included in this study, and subsequent studies can further analyze these in depth. In addition, biomarkers, such as WDR72 and methylation of LMX1A, have been reported to be closely associated with BCa recurrence [30, 31]. These factors were not included in this study, and we will try to build more efficient predictive models by combining clinical factors, radiomics, and important biomarkers. What's more, by repeatedly modeling and using multiple data dimensionality reduction methods to reduce overfitting, it is difficult to find an absolute subset of the selected features because it relies on the segmentation of the training and test sets. We believe that future research may be able to establish a variety of models and provide the more reliable solution for clinical diagnosis and treatment after multiple evaluations. As a result, this study still needs to be further improved before clinical application [32].

In conclusion, this study combined clinical factors and radiomics characteristics of CT to construct a composite model to predict local or metastatic recurrence of BCa after RC. These preliminary results showed that the model has good efficacy in stratifying recurrence risk, which may provide some help for the individualized treatment of BCa patients.

## Data Availability

The data sets used and analyzed in this study are available from the respective authors if reasonably requested.

## Abbreviations

BCa: Bladder cancer
RC: Radical cystectomy
CT: Computed tomography
DCA: Decision curve analyses
AUC: Area under the curve
CI: Confidence interval
MIBC: Muscle invasive bladder cancer
NMIBC: Non-muscle invasive bladder cancer
NMMIBC: Nonmetastatic muscle invasive bladder cancer
HRNMIBC: High risk non-muscle invasive bladder cancer
MRI: Magnetic resonance imaging
ROI: Region of interest
GLCM: Gray-level cooccurrence matrix
GLRLM: Gray-level run length matrix
GLSZM: Gray-level size zone matrix
NGTDM: Neighborhood gray tone difference matrix
GLDM: Gray-level dependence matrix
ICC: Interclass correlation coefficient
ROC: Receiver operating characteristic curve
KM: Kaplan–meier
RFS: Recurrence free survive
